# Aminoacyl transfer ribonucleic acid synthetase complex-interacting multifunctional protein 1 induces microglial activation and M1 polarization *via* the mitogen-activated protein kinase/nuclear factor-kappa B signaling pathway

**DOI:** 10.3389/fncel.2022.977205

**Published:** 2022-09-07

**Authors:** Yebin Oh, Hak-Jun Jung, Seungwon Hong, Yerim Cho, Jiyeong Park, Daeho Cho, Tae Sung Kim

**Affiliations:** ^1^Department of Life Sciences, College of Life Sciences and Biotechnology, Korea University, Seoul, South Korea; ^2^Institute of Convergence Science, Korea University, Seoul, South Korea

**Keywords:** AIMP1, microglia, neuroinflammation, MAPK, NF-κB

## Abstract

Activation of microglia, which is the primary immune cell of the central nervous system, plays an important role in neuroinflammation associated with several neuronal diseases. Aminoacyl tRNA synthetase (ARS) complex-interacting multifunctional protein 1 (AIMP1), a structural component of the multienzyme ARS complex, is secreted to trigger a pro-inflammatory function and has been associated with several inflammatory diseases. However, the effect of AIMP1 on microglial activation remains unknown. AIMP1 elevated the expression levels of activation-related cell surface markers and pro-inflammatory cytokines in primary and BV-2 microglial cells. In addition to the AIMP1-mediated increase in the expression levels of M1 markers [interleukin (IL)-6, tumor necrosis factor-α, and IL-1β], the expression levels of CD68, an M1 cell surface molecule, were also increased in AIMP-1-treated microglial cells, while those of CD206, an M2 cell surface molecule, were not, indicating that AIMP1 triggers the polarization of microglial cells into the M1 state but not the M2 state. AIMP1 treatment induced the phosphorylation of mitogen-activated protein kinases (MAPKs), while MAPK inhibitors suppressed the AIMP1-induced microglial cell activation. AIMP1 also induced the phosphorylation of the nuclear factor-kappa B (NF-κB) components and nuclear translocation of the NF-κB p65 subunit in microglial cells. Furthermore, c-Jun N-terminal kinase (JNK) and p38 inhibitors markedly suppressed the AIMP1-induced phosphorylation of NF-κB components as well as the nuclear translocation of NF-κB p65 subunit, suggesting the involvement of JNK and p38 as upstream regulators of NF-κB in AIMP1-induced microglial cell activation. The NF-κB inhibitor suppressed the AIMP1-induced M1 polarization of the microglial cells. Taken together, AIMP1 effectively induces M1 microglial activation *via* the JNK and p38/NF-κB-dependent pathways. These results suggest that AIMP1 released under stress conditions may be a pathological factor that induces neuroinflammation.

## Introduction

Aminoacyl tRNA synthetases (ARSs) are crucial enzymes that link amino acids to their corresponding transfer RNAs (tRNAs) to produce proteins (Zhou et al., 2020). ARS complex-interacting multifunctional protein 1 (AIMP1) is a core auxiliary protein that maintains the assembly of the ARS complex (Park et al., 2010). Under various conditions, such as cellular stress, AIMP1 is released from the ARS complex and secreted as a pro-inflammatory cytokine (Liang et al., 2015). Patients with systemic lupus erythematosus (SLE) have significantly higher levels of serum AIMP1 than healthy control (Ahn et al., 2018). In addition, compared to healthy individuals, patients with rheumatoid arthritis, an inflammatory disease, exhibit higher inflammatory cytokine, and AIMP1 levels in the peripheral blood and synovial fluid (Hong et al., 2015). Secreted AIMP1 activates the extracellular signal-regulated kinase (ERK)-1/2 to increase the release of tumor necrosis factor (TNF)-α (Kwon et al., 2012). Previously, we demonstrated that AIMP1 activates the nuclear factor-kappa B (NF-κB) pathway in bone marrow-derived dendritic cells and macrophages, thereby promoting Th1 responses (Kim et al., 2006, 2008).

Microglia, the primary immune cell of the brain, is the tissue-resident macrophage of the central nervous system (CNS) (Bennett and Bennett, 2020). Various immune cells, including microglia, interact with the resident cells. Microglia is involved in homeostasis and defense mechanisms against pathological invasion (Hickman et al., 2018). Immune responses lean toward either pole of the immune equilibrium under pathological conditions, whereas they are fine-tuned in the normal state (Tang and Le, 2016). In response to injurious stimuli, such as damage-associated molecular patterns (DAMPs) and pathogen-associated molecular patterns (PAMPs), the microglia generates various pro-inflammatory factors (Glass et al., 2010). According to the stimulated condition, the microglial state is classified into classical activation, alternative activation, and acquired deactivation states (Colton, 2009). Microglia with a classical activation state is labeled as M1 microglia, while those with other states are labeled as M2 microglia. M1 microglia secretes pro-inflammatory factors and cytokines that are closely linked to neuroinflammation and cause several neuronal diseases (Wang et al., 2015). In contrast to pro-inflammatory M1 cells, alternatively activated M2 microglia suppresses inflammation and restores homeostasis (Cherry et al., 2014).

Diverse signaling pathways associated with cytokines and inflammation regulates the microglial phenotypic polarization (Li et al., 2021). The mitogen-activated protein kinase (MAPK) signaling pathway, which consists of ERK, c-Jun N-terminal kinase (JNK), and p38, is a major kinase family involved in various inflammatory processes (Rao, 2001). The MAPK pathway regulates the expression levels of various pro-inflammatory cytokines and factors, such as inducible nitric oxide synthase, cyclooxygenase-2, interleukin (IL)-6, and tumor necrosis factor-α (Lim et al., 2018). The activated NF-κB signaling pathway is associated with classic inflammation, which promotes M1 microglial polarization (Saijo and Glass, 2011). During neuronal injury and death of the CNS, endogenous or exogenous PAMPs stimulate Toll-like receptor 4 (TLR4) in microglia, thereby activating the NF-κB signaling pathway (Kumar, 2019). TLR4/NF-κB signaling induces the generation of pro-inflammatory cytokines and neuroinflammation (Chen et al., 2019).

As the microglia-mediated neuroinflammation is the major cause of several neuronal diseases, it is necessary to investigate the direct effects of AIMP1 on microglial activation and polarization (Streit et al., 2004). In the present study, we determined the effects of AIMP1 on the activation and polarization of microglia, the brain resident macrophages, as well as the involvement of MAPKs and NF-κB in the underlying mechanism.

## Materials and methods

### Mice

Postnatal 3–5-day C57/BL6 mice (Young Bio, Osan city, South Korea) were used for this study. The animals were kept in a specific pathogen-free facility, and the experiments were performed according to the guidelines of the Korea University Institutional Animal Care and Use Committee (KUIACUC-2021-0094).

### BV-2 cell line and recombinant Aminoacyl transfer ribonucleic acid synthetase complex-interacting multifunctional protein 1

The murine microglial cell line, BV-2 cells, were cultured in DMEM with 4.5 g/L glucose, L-glutamine, sodium pyruvate (Corning, 10-013-CVR), with 5% FBS, 100 U/ml penicillin, and 100 μg/ml streptomycin. Recombinant AIMP1 was overexpressed as a His-tag fusion protein in IPTG-induced Escherichia coli BL21 (DE3) and purified by nickel affinity chromatography, followed by a HiTrap Q column (GE Healthcare, 17-5156-01) for anion exchange chromatography. The eluent was further purified by gel filtration chromatography using Superdex75 16/600 (GE Healthcare, 28-9893-33) to remove residual LPS. The endotoxin level in each purification lot was determined using a ToxinSensorTM chromogenic LAL endotoxin assay kit (Genscript, Nanjing, China). Lots containing < 0.05 EU/μg protein were used for this study.

### Isolation of the primary microglial cells

Primary microglial cells were obtained from mixed glial cultures prepared from the cerebral cortexes of postnatal 3–5-day mice. Briefly, C57BL/6 mice were sacrificed, and the brains were dissected in cold PBS. The cortexes excluding meninges were enzymatically digested and mechanically dissociated using Neural Tissue Dissociation Kit (Miltenyi Biotec, 130-092-628). The filtered single-cell suspension was diluted in Dulbecco’s Modified Eagle’s Medium (DMEM) with 4.5 g/L glucose, L-glutamine, and sodium pyruvate (Corning, 10-013-CVR), with 10% fetal bovine serum (FBS; Gibco, 16000-044), 10 ng/ml GM-CSF (ProSpec, cyt-222), 100 U/ml penicillin and 100 μg/ml streptomycin (Corning, 30-002-CI). Cells of 2∼3 pups were plated into a tissue culture flask and cultured in a humidified atmosphere containing 5% CO_2_ at 37°C. The next day, the medium was changed to remove debris. On day 10∼14, the culture flasks were shaken on the 1-dimensional shaker at 200 rpm for 5 min and vigorously tapped to collect microglial cells. The non-adherent cells after shaking were harvested and used for experiments. The purity of CD11b^+^CD45^low^ primary microglia was determined by flow cytometry (> 85%).

### Flow cytometric analysis

The microglial cells (1 × 10^6^ cells/well) were washed with PBS and harvested with the trypsin/EDTA. Subsequently, the reaction was stopped with culture media and washed with FACS buffer (0.5% FBS and filtered 0.05% NaN_3_ in PBS). The cells were blocked for 15 min at room temperature with the mouse IgG then stained with APC-conjugated anti-mouse CD11b (BD Pharmingen™, 561690) and the PE-conjugated anti-mouse antibodies: CD40 (BD Pharmingen™, 553791), CD45 (BD Pharmingen™, 553081), and CD86 (BD Pharmingen™, 553692).

### Reverse transcription-polymerase chain reaction

The mRNA was obtained from the microglial cells (1 × 10^6^ cells/well) using the RiboEX reagent (GeneAll Biotechnology, Seoul, South Korea) according to the manufacturer’s protocol. It was reverse transcribed into the cDNA with CycleScriptTM reverse transcriptase (Bioneer) and amplified by the PCR. After the PCR amplification, the products were separated on the 2% agarose gels and stained with ethidium bromide.

### Enzyme-linked immunosorbent assay

The concentrations of IL-1β, IL-6, and TNF-α in the supernatants were measured in triplicate using ELISA kits (eBioscience, San Diego, CA, United States). The wells were finally washed with PBST (0.05% Tween-20 in PBS), and *o*-phenylenediamine containing citrate and H_2_O_2_ was added to each well and incubated for 15 min at room temperature. To stop the reaction, 2N H_2_SO_4_ was added to each well. Developed colors were detected on a VMax kinetic microplate reader at 490 nm.

### Immunofluorescent microscopy

The microglial cells (1 × 10^6^ cells/well) were fixed with 4% paraformaldehyde and blocked with 0.1% Triton X-100 and 0.5% BSA in PBS for 30 min at room temperature. The cells were incubated with mouse anti-CD68 (1:100 dilution) or rabbit anti-CD206 (1:200 dilution), or rabbit anti-NF-κB p65 (1:300 dilution) at 4°C overnight, followed by staining with Alexa Fluor 488-conjugated anti-rabbit IgG antibody (1:300 dilution) or Alexa Fluor 594-conjugated anti-mouse IgG antibody (1:200 dilution) for 1 h at the room temperature. Nuclei were counterstained with DAPI (Molecular Probes, 3 μM) for 3 min and observed with a confocal laser scanning microscope (LSM 700, Carl Zeiss, Oberkochen, Germany).

### Western blot analysis

The microglial cells (1 × 10^6^ cells/well) were harvested at the indicated time points, washed with PBS, and lysed in RIPA buffer containing protease and phosphatase inhibitor cocktail. The whole-cell lysates were then separated on 10% SDS-PAGE and transferred to PVDF membranes. The membranes were blocked with 5% skim milk for 1 h and incubated overnight at 4°C with respective antibodies against phosphorylated p38, JNK, ERK, IKKβ, IκB-α, NF-κB p65, or GAPDH. The membranes were then treated with HRP-conjugated anti-mouse IgG or anti-rabbit IgG at room temperature. The bands were visualized with chemiluminescent HRP substrate (Millipore Corporation, Billerica, MA, United States) and an X-Ray film processor (JP-33, JPI, Seoul, South Korea).

### Statistical analyses

Statistical significance was estimated by unpaired Student’s *t*-test or one-way analysis of variance (ANOVA) or two-way ANOVA with a Bonferroni post-test. To compare experimental groups, an unpaired Student’s *t*-test was conducted in SigmaPlot version 10.0 software (Systat Software Inc., Washington, CA, United States). One-way analysis of variance (ANOVA) or two-way ANOVA with a Bonferroni post-test was performed in IBM SPSS Statistic 25 software (IBM, New York City, NY, United States) for multiple comparisons. The data were represented as the mean ± SD of the three independent experiments. The *p*-values ≤ 0.05 were regarded as statistically significant.

## Results

### Aminoacyl transfer ribonucleic acid synthetase complex-interacting multifunctional protein 1 induces the activation of primary microglial cells

**FIGURE 1:**
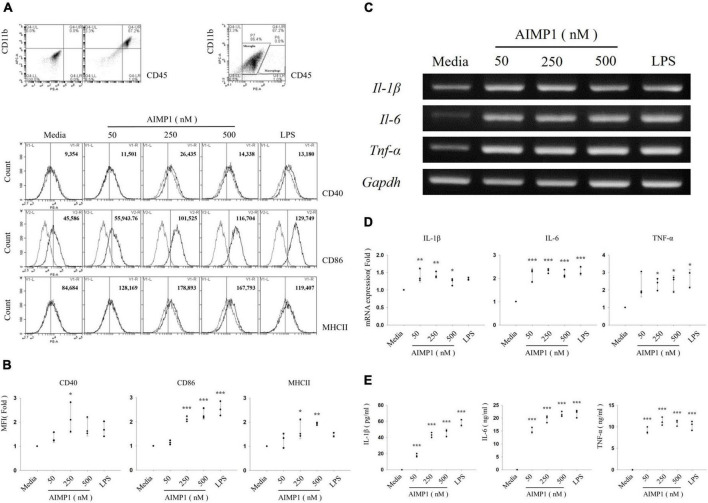
Aminoacyl tRNA synthetase (ARS)-interacting multifunctional protein 1 (AIMP1) elevates the expression levels of surface markers and pro-inflammatory cytokines in the primary microglia Primary microglial cells were obtained from the cerebral cortex of C57BL/6 mice and cultured with AIMP1 (50, 250, and 500 nM) or lipopolysaccharides (LPSs) (100 ng/mL) for 24 **(A,B,E)** or 6 h **(C,D)**. **(A)** The values of histograms signify the mean fluorescence intensity (MFI) of CD40, CD86, and MHC II in CD11b^+^CD45^low^ gated microglia. **(B)** Scatter plots represent the fold ratio based on the MFI of each surface marker in the medium group. The data represent the mean ± standard deviation (SD) values of three independent experiments. **(C)** mRNA expression levels of interleukin (IL)-1β, IL-6, and tumor necrosis factor (TNF)-α were quantified using reverse transcription-polymerase chain reaction (RT-PCR). Glyceraldehyde-3-phosphate dehydrogenase (GAPDH) served as a control. **(D)** Scatter plots represent the fold ratio of mRNA expression in each group compared with the medium group. The data represent the mean ± SD of three independent experiments. **(E)** Secretion of pro-inflammatory cytokines was analyzed using an enzyme-linked immunosorbent assay (ELISA). Scatter plots show the mean ± SD of three independent experiments performed in triplicate. Statistical significance was evaluated using one-way analysis of variance (ANOVA) with a Bonferroni *post hoc* test for multiple comparisons; *P < 0.05, **P < 0.01, and ***P < 0.001 compared to the medium group.

Expression levels of the major histocompatibility complex (MHC) and costimulatory molecules are increased upon the activation of resting microglial cells (Yang et al., 2010). Therefore, to determine whether AIMP1 affects microglial activation, primary microglial cells isolated from the cerebral cortices of C57BL/6 mice were cultured for 24 h with varying concentrations of AIMP1, and the expression levels of activation-related surface markers were determined using cytometric analysis. AIMP1 significantly increased the expression of CD40, CD86, and MHC II on CD11b + CD45low gated primary microglial cells (Figures 1A,B). The expression levels of CD86 and MHC II were upregulated by AIMP1 in a dose-dependent manner (Figure 1B).

As activated microglial cells release pro-inflammatory cytokines that can mediate neuronal damage (Dheen et al., 2007), we assessed the mRNA levels of pro-inflammatory cytokines, such as interleukin (IL)-1β, IL-6, and TNF-α, in primary microglial cells. All doses of AIMP1 increased the mRNA expression levels of IL-1β, IL-6, and TNF-α in primary microglial cells (Figures 1C,D). In addition, the secretion of pro-inflammatory cytokines by AIMP1-treated or untreated primary microglial cells was analyzed using enzyme-linked immunosorbent assays (ELISAs) specific for each cytokine. The secreted levels of all three pro-inflammatory cytokines were upregulated in AIMP1-treated microglial cells compared to those in untreated cells. In particular, the secretion of IL-1β and IL-6 increased in proportion to the dose of AIMP1 (Figure 1E). Taken together, these results demonstrate that AIMP1 induces the activation of primary microglial cells.

### Aminoacyl transfer ribonucleic acid synthetase complex-interacting multifunctional protein 1 induces M1 activation of microglial cells

Microglial cells commonly acquire pro-inflammatory M1 or anti-inflammatory M2 phenotypes, contingent upon factors that stimulate their activation (Tang and Le, 2016). As AIMP1 strongly increased the expression of pro-inflammatory cytokines (IL-1β, IL-6, and TNF-α) in this study, AIMP1 treatment is likely to polarize microglial cells to the M1 phenotype. To confirm whether AIMP1 induces M1 microglial polarization, the primary microglia were treated with AIMP1, and the expression of CD68, an M1 cell surface marker, and CD206, an M2 cell surface marker, were determined using confocal microscopy. As shown, AIMP1 increased the expression levels of CD68 in primary microglia, whereas the expression levels of CD206 were not (Supplementary Figures 1A,B). These results demonstrate that AIMP1 triggers the polarization of microglial cells to the M1 state but not the M2 state.

**FIGURE 2:**
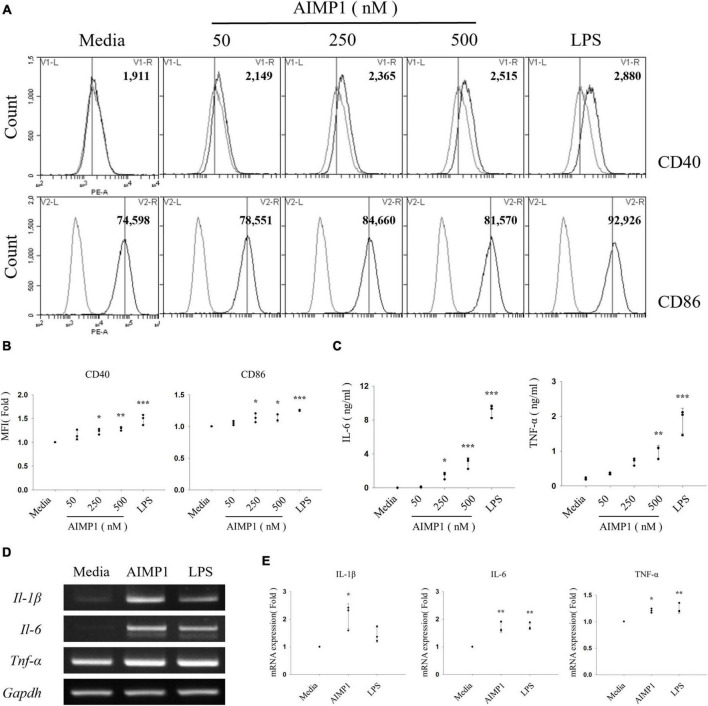
ARS complex-interacting multifunctional protein 1 (AIMP1) increases the expression levels of surface markers and pro-inflammatory cytokines in BV-2 microglial cells BV-2 cells were cultured with AIMP1 or LPS (100 ng/mL) for 24 **(A–C)** and with AIMP1 (250 nM) for 6 h **(D,E)**. **(A)** The values of histograms indicate the MFI of CD40 and CD86 in CD11b^+^ -gated microglia. **(B)** Scatter plots represent the fold ratio based on the MFI of each surface marker in the medium group. The data represent the mean ± SD of three independent experiments. **(C)** Secretion of IL-6 and TNF-α was analyzed using ELISA. Scatter plots show the mean ± SD of three independent experiments performed in triplicate. **(D)** mRNA expression levels of pro-inflammatory cytokines were quantified using RT-PCR. GAPDH served as a control. **(E)** Scatter plots represent the fold ratio of mRNA expression in each group compared to that in the medium group. The data represent the mean ± SD of three independent experiments. Statistical significance was evaluated using one-way ANOVA with a Bonferroni *post hoc* test for multiple comparisons; *P < 0.05, **P < 0.01, and ***P < 0.001 compared to the medium group.

Next, to determine whether AIMP1 exhibits a similar enhancing effect on the activation of BV-2 cells, an immortalized murine microglial cell line, the cells were treated with AIMP1, and the expression levels of the activation cell surface markers were determined using cytometric analysis. AIMP1 upregulated the expression levels of CD40 and CD86 in in CD11b^+^ -gated BV-2 cells, similar to that in primary microglial cells (Figures 2A,B). CD40 expression levels increased in a dose-dependent manner following AIMP1 treatment (Figure 2B). Additionally, the secretion of IL-6 and TNF-α in BV-2 cells increased in proportion to the AIMP1 dose (Figure 2C). Likewise, the mRNA expression levels of IL-1β, IL-6, and TNF-α were elevated in BV-2 cells (Figures 2D,E). Overall, AIMP1 induced the activation of both BV-2 cells and primary microglial cells.

Furthermore, to exclude a possibility that endotoxin contamination in the AIMP1 protein may affect the AIMP1-mediated activation and M1 polarization of microglial cells, BV-2 cells were cultured for 24 h with AIMP1 in the absence or presence of polymyxin B (PMB) to inhibit lipopolysaccharide (LPS) signaling. Treatment of BV-2 cells with PMB significantly inhibited the LPS-induced increase in the expression levels of pro-inflammatory cytokines. However, PMB treatment did not affect the AIMP1-mediated expression of pro-inflammatory cytokines (Supplementary Figure 2A). In addition, AIMP was first boiled for 30 min at 100°C and then used to treat BV-2 cells. AIMP1-mediated microglial activation was reduced by boiling, whereas LPS-induced BV-2 cell activation remained unchanged (Supplementary Figure 2B). Moreover, less than 0.05 endotoxin unit/μg of protein (1 endotoxin unit = 0.1 ng/mL Escherichia coli LPS) was detected, as determined by the Limulus amebocyte lysate assay. These endotoxin concentrations are insufficient for microglial activation. These results indicate that AIMP1 increases the expression levels of activation-related cell surface markers and pro-inflammatory cytokines in primary microglia and microglial cell lines and that these effects are not due to LPS contamination.

### c-Jun N-terminal kinase and p38 mitogen-activated protein kinases are involved in the Aminoacyl transfer ribonucleic acid synthetase complex-interacting multifunctional protein 1-mediated activation of BV-2 microglial cells

**FIGURE 3:**
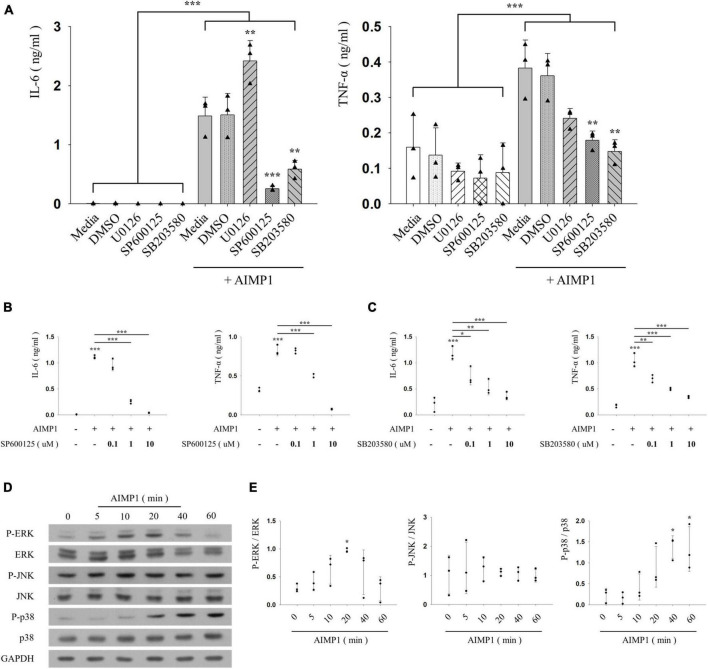
ARS complex-interacting multifunctional protein 1 (AIMP1) activates BV-2 cells *via* the c-Jun N-terminal kinase (JNK) and p38 pathways **(A–C)** BV-2 cells were pre-treated with the mitogen-activated protein kinase (MAPK) inhibitors for 30 min, and subsequently with AIMP1 (250 nM) for 24 h. The secretion of IL-6 and TNF-α is analyzed by ELISA. Bar and scatter graphs show the mean ± SD of three independent experiments. Statistical significance was evaluated using two-way **(A)** and one-way ANOVA **(B,C)** with a Bonferroni *post hoc* test for multiple comparisons. **(D,E)** BV-2 cells were incubated with AIMP1 (250 nM) in a time-dependent manner. **(D)** ERK, JNK, p38, and their phosphorylated forms are quantified using western blotting analysis. Glyceraldehyde-3-phosphate dehydrogenase (GAPDH) served as a control. **(E)** Scatter plots represent the phosphorylation ratio of MAPK proteins. It shows the mean ± SD of three independent experiments. Statistical significance was evaluated using one-way ANOVA with a Bonferroni *post hoc* test for multiple comparisons; *P < 0.05, **P < 0.01, and ***P < 0.001 compared to the medium group.

Inflammatory activation of microglia is regulated by representative intracellular signaling pathways, such as the MAPK signaling pathway (Spencer et al., 2012). To investigate the involvement of MAPKs in AIMP1-mediated microglial activation, BV-2 cells were pre-treated with each inhibitor of three canonical MAPK subfamilies (U0126 for ERK, SP600125 for JNK, and SB203580 for p38), after which they were incubated with AIMP1, and the secretion levels of pro-inflammatory cytokines were determined using ELISA. Among these inhibitors, SP600125 and SB203580 suppressed the secretion of IL-6 and TNF-α in a dose-dependent manner, whereas U0126 exerted little effects (Figures 3A–C). Moreover, the levels of phosphorylation and expression of each MAPK were determined in AIMP1-treated BV-2 microglial cells using western blotting analysis. AIMP1 triggered the phosphorylation of ERK and p38 in a time-dependent manner. JNK phosphorylation was relatively high in untreated BV-2 cells and remained unchanged in the presence of AIMP1 (Figures 3D,E). Consequently, these results indicate that AIMP1 induces the activation of BV-2 cells *via* the JNK-and p38 MAPK-dependent signaling pathways.

### The nuclear factor-kappa B signaling pathway contributes to the aminoacyl transfer ribonucleic acid synthetase complex-interacting multifunctional protein 1-mediated activation of BV-2 microglial cells

**FIGURE 4:**
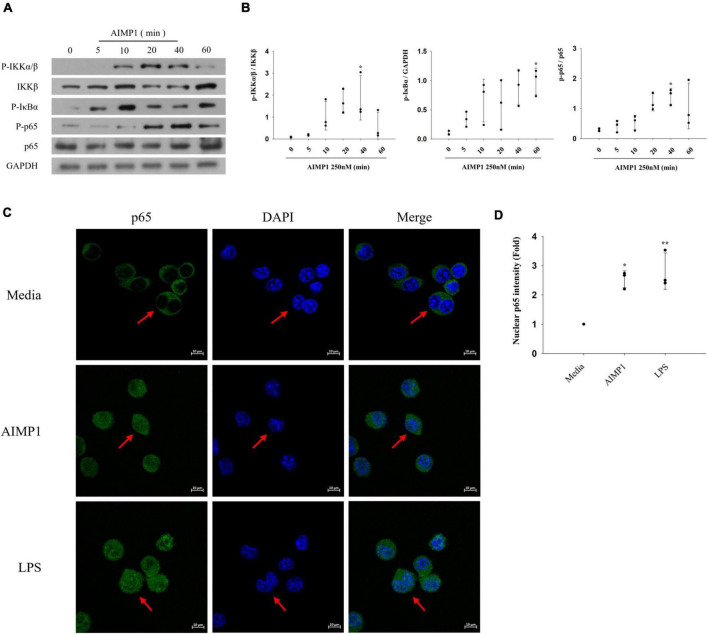
ARS complex-interacting multifunctional protein 1 (AIMP1) activates BV-2 cells *via* the nuclear factor-kappa B (NF-κB) pathway **(A,B)** BV-2 cells were incubated with AIMP1 (250 nM) in a time-dependent manner. IKKβ, p65, and phosphorylated forms of IKKα/β, IκB-α, p65 were quantified by western blotting analysis. GAPDH served as a control. **(B)** Scatter plots represent the phosphorylation ratio of NF-κB proteins. It shows the mean ± SD of three independent experiments. **(C,D)** Translocation of NF-κB p65 into the nucleus was observed by confocal microscopy. The representative data are shown. **(D)** Scatter plots represent the fold ratio based on the fluorescence intensity of nuclear p65 in the medium group. It shows the mean ± SD of three independent experiments. Statistical significance was evaluated using one-way ANOVA with a Bonferroni *post hoc* test for multiple comparisons; *P < 0.05 and **P < 0.01 compared to the medium group.

Among several representative signaling pathways that progress during microglial activation, the NF-κB signaling pathway triggers the production of inflammatory mediators, thereby intensifying neuroinflammation (Dresselhaus and Meffert, 2019). To further explore the additional signaling components involved in AIMP1-mediated microglial activation, BV-2 cells were treated with AIMP1, and the phosphorylated forms of IkappaB kinase (IKK)α/β, IκB-α, and p65 were quantified using western blotting analysis. AIMP1 induced the phosphorylation of IKKα/β, IκB-α, and p65 in BV-2 cells over time (Figures 4A,B). Furthermore, nuclear translocation of the NF-κB p65 subunit was observed in AIMP1-treated BV-2 cells (Figures 4C,D), indicating that, in addition to JNK and p38 MAPKs, activation of the NF-κB pathway occurs in AIMP1-treated BV-2 cells.

### Aminoacyl transfer ribonucleic acid synthetase complex-interacting multifunctional protein 1 induces the activation of microglial cells *via* the c-Jun N-terminal kinase and p38/nuclear factor-kappa B signaling pathways

**FIGURE 5:**
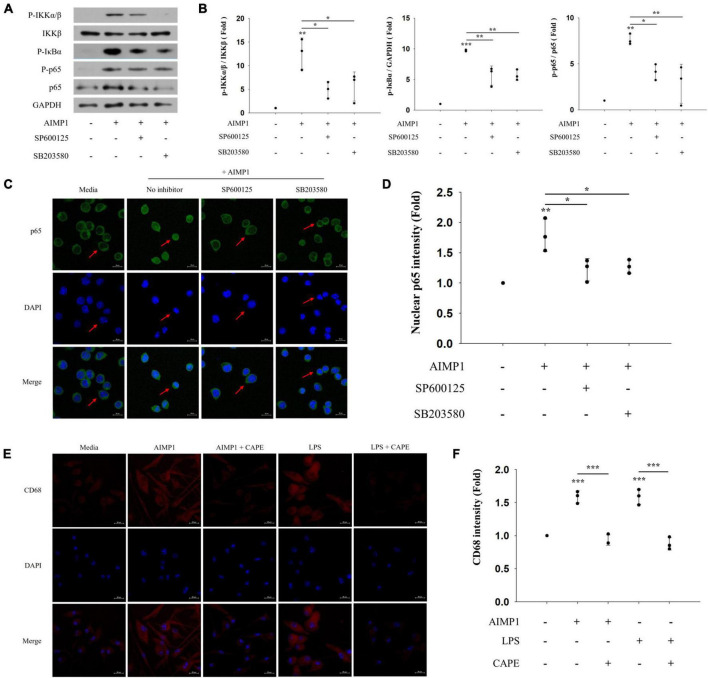
c-Jun N-terminal kinase (JNK) and p38 act as upstream regulators of NF-κB during AIMP-induced microglial activation Following pretreatment with 1 μM of the MAPK inhibitors (SP600125 and SB203580) for 30 min, BV-2 cells were treated with AIMP1 (250 nM) for 40 min **(A,B)** or 1 h **(C,D)**. **(A)** IKKβ, p65, and phosphorylated forms of IKKα/β, IκB-α, and p65 were quantified using western blot analysis. GAPDH served as a control. **(B)** Scatter plots represent the phosphorylation ratio of NF-κB proteins. It shows the mean ± SD of three independent experiments. **(C,D)** Transflocation of NF-κB p65 into the nucleus was observed using confocal microscopy. **(D)** Scatter plots represent the fold ratio based on the fluorescence intensity of nuclear p65 in the medium group. It shows the mean ± SD of three independent experiments. **(E,F)** Following pretreatment with the NF-κB inhibitor (CAPE) for 30 min, the primary microglia were cultured with AIMP1 (250 nM) for 72 h. The expression of CD68 in the primary microglia was observed using confocal microscopy. The representative data are shown. **(F)** Scatter plots represent the fold ratio based on the fluorescence intensity of CD68 in the medium group. It shows the mean ± SD of three independent experiments. Statistical significance was evaluated using one-way ANOVA with a Bonferroni *post hoc* test for multiple comparisons; *P < 0.05, **P < 0.01, and ***P < 0.001 compared to the medium group.

As the activation of NF-κB can be caused by MAPK signaling pathways (Qin et al., 2018), we determined whether the JNK and p38 pathways also regulate the NF-κB signaling pathway during AIMP1-induced microglial activation. AIMP1-induced phosphorylation of IKKα/β, IκB-α, and p65 was reduced after pretreatment with JNK and p38 inhibitors (Figures 5A,B). Similarly, the translocation of NF-κB p65 into the nucleus of BV-2 cells was suppressed by the inhibition of JNK and p38 signaling (Figures 5C,D). Therefore, these results demonstrate that JNK and p38 MAPKs act as upstream regulators of the NF-κB pathway during the AIMP1-induced activation of BV-2 cells.

In addition, we further examined the involvement of the downstream NF-κB pathway in AIMP1-mediated polarization of microglial cells. AIMP1-induced M1 polarization of the primary microglia was suppressed after pretreatment with an NF-κB inhibitor (CAPE) (Figures 5E,F). Thus, AIMP1 activates and polarizes the microglial cells to the M1 phenotype *via* the JNK and p38/NF-κB signaling pathways.

## Discussion

Aminoacyl transfer ribonucleic acid synthetase complex-interacting multifunctional protein 1 is secreted from stressed or dying cells exposed to cellular stress-inducing conditions (Ko et al., 2001) and exhibits a variety of cytokine-like functions, including anti-angiogenic and pro-inflammatory activities (Park et al., 2002). Previously, we demonstrated that AIMP1 activated various immune cells, including monocytes, macrophages, and dendritic cells (DCs) *via* three MAPKs (ERK1/2, JNK, and p38) and the NF-κB pathway (Ko et al., 2001; Kim et al., 2006, 2008). However, it is necessary to investigate the role of AIMP1 in the activation and polarization of microglia, tissue-resident immune cells of the CNS, as microglial polarization is associated with several neuronal diseases resulting from neuroinflammation and neuronal cell death under cellular stress. This study is the first to demonstrate that AIMP1 activates microglia and polarizes them to the pro-inflammatory M1 phenotype *via* the MAPK–NF-κB signaling pathway.

M1 polarization of microglial cells is closely linked to the development and progression of neuroinflammation and several neuronal diseases. In this study, we demonstrated that AIMP1 significantly increased the expression levels of pro-inflammatory M1 cytokines, such as IL-1β, IL-6, and TNF-α. Furthermore, the expression levels of the M1 surface marker were significantly elevated in AIMP1-treated microglial cells, whereas the expression levels of the M2 surface marker remained unchanged. TNF-α induces AIMP1 expression and secretion from immune cells, including macrophages, which are recruited to the wounded regions of the skin (Kim et al., 2008). As AIMP1 can induce the expression of TNF-α in immune cells, AIMP1 and TNF-α appear to form a positive feedback loop with one another that amplifies their inflammatory response to tissue injury.

Aminoacyl transfer ribonucleic acid synthetase complex-interacting multifunctional protein 1 may induce M1 microglial activation *via* NF-κB activation through the JNK and p38 MAPK signaling pathways. Intracellular signaling pathways, including NF-κB and MAPK pathways, are associated with the regulation of pro-inflammatory cytokines following stimulation of several immune cells, such as macrophages and DCs (Liu et al., 2017; Dorrington and Fraser, 2019). In this study, we demonstrated that AIMP1 increases the phosphorylation of ERK and p38, but not JNK, in BV-2 microglial cells. Phosphorylated JNK levels were relatively high in untreated BV-2 cells and remained unchanged after AIMP1 treatment. Among the three major MAPK components, pharmacological inhibition of JNK and p38 reduced the secretion of AIMP1-induced pro-inflammatory cytokines in a dose-dependent manner, while the ERK inhibitor showed the opposite trend, indicating the minor contribution of ERK phosphorylation to AIMP1-mediated microglial activation. Furthermore, AIMP1-induced phosphorylation of IKKα/β, IκB-α, and p65, and subsequent nuclear translocation of p65 indicate the contribution of the NF-κB pathway in AIMP1-induced activation of microglia. Finally, inhibition of JNK and p38 MAPK alleviates these effects, and inhibition of NF-κB signaling avoids the M1 polarization of the microglia, indicating that JNK and p38 MAPKs are upstream regulators of the NF-κB pathway during AIMP-mediated activation of microglia.

Although a receptor for AIMP1 has not been reported, AIMP1 may interact with the cell surface to induce microglial activation and polarization, as exogenous proteins generally cannot pass through the cell membrane with amphipathic polarity. Previously, we reported that AIMP1 regulates TCR signaling by interfering with lipid raft association (Kim et al., 2019). We also confirmed the localization of TRS, an ARS, on the surface of DCs, as demonstrated by immunofluorescence microscopic analysis (Jung et al., 2020). Lysyl-tRNA synthetase, another ARS, interacts with the 67-kDa laminin receptor, facilitating cancer cell migration during metastasis (Cho et al., 2014; Kim et al., 2014).

Overall, our results suggest the possible role of AIMP1 in neuroinflammation *via* M1 microglial activation. As AIMP1 exists in the neuronal cells of diverse CNS sections, comprising the spinal cord and hippocampus (Zhu et al., 2009), it can be secreted from cells under cellular stress-inducing conditions and may contribute to the progression of neuroinflammatory diseases. Secreted AIMP1 is detected in the serum of patients with inflammatory diseases, including SLE (Ahn et al., 2018). Thus, further studies on the detailed mechanism underlying AIMP1-induced microglial activation and polarization are required to develop novel therapeutic strategies for neuroinflammation. Furthermore, it is important to investigate the effects of AIMP1 on the pathological roles of amyloid-β, α-synuclein, and tau, which are the well-known causative factors of neuroinflammation and neuronal diseases (Gao et al., 2011; Ismail et al., 2020).

## Data availability statement

The datasets presented in this study can be found in online repositories. The names of the repository/repositories and accession number(s) can be found in the article/Supplementary material.

## Ethics statement

The animal study was reviewed and approved by Korea University Institutional Animal Care and Use Committee (KUIACUC-2021-0094).

## Author contributions

YO designed and performed all the experiments. YO, HJ, SH, YC, and JP contributed to acquiring the experimental results. YO, HJ, and TK analyzed and interpreted the experimental results. YO and TK collaborated on manuscript writing. TK and DC supervised the study and corrected the manuscript. All authors read and approved the final manuscript.

## References

[B1] Ahn S. S. Hong S. H. Park Y. Jung S. M. Song J. J. Park Y. B. (2018). Serum aminoacyl-tRNA synthetase-interacting multifunctional protein-1 (AIMP1), a novel disease activity predictive biomarker of systemic lupus erythematosus. *Clin. Exp. Rheumatol.* 36 533–539. 29352840

[B2] Bennett M. L. Bennett F. C. (2020). The influence of environment and origin on brain resident macrophages and implications for therapy. *Nat. Neurosci.* 23 157–166. 10.1038/s41593-019-0545-6 31792468

[B3] Chen C. Chu S. F. Ai Q. D. Zhang Z. Guan F. F. Wang S. S. (2019). CKLF1 Aggravates focal cerebral ischemia injury at early stage partly by modulating microglia/macrophage toward M1 polarization through CCR4. *Cell. Mol. Neurobiol.* 39 651–669. 10.1007/s10571-019-00669-5 30982091 PMC11462892

[B4] Cherry J. D. Olschowka J. A. O’Banion M. K. (2014). Neuroinflammation and M2 microglia: The good, the bad, and the inflamed. *J. Neuroinflammation* 11:98. 10.1186/1742-2094-11-98 24889886 PMC4060849

[B5] Cho H. Y. Ul Mushtaq A. Lee J. Y. Kim D. G. Seok M. S. Jang M. (2014). Characterization of the interaction between lysyl-tRNA synthetase and laminin receptor by NMR. *FEBS Lett.* 588 2851–2858. 10.1016/j.febslet.2014.06.048 24983501

[B6] Colton C. A. (2009). Heterogeneity of microglial activation in the innate immune response in the brain. *J. Neuroimmune Pharmacol.* 4 399–418. 10.1007/s11481-009-9164-4 19655259 PMC2773116

[B7] Dheen S. T. Kaur C. Ling E. A. (2007). Microglial activation and its implications in the brain diseases. *Curr. Med. Chem.* 14 1189–1197. 10.2174/092986707780597961 17504139

[B8] Dorrington M. G. Fraser I. D. C. (2019). NF-κB signaling in macrophages: Dynamics, crosstalk, and signal integration. *Front. Immunol.* 10:705. 10.3389/fimmu.2019.00705 31024544 PMC6465568

[B9] Dresselhaus E. C. Meffert M. K. (2019). Cellular specificity of NF-κB function in the nervous system. *Front. Immunol.* 10:1043. 10.3389/fimmu.2019.01043 31143184 PMC6520659

[B10] Gao H. M. Zhang F. Zhou H. Kam W. Wilson B. Hong J. S. (2011). Neuroinflammation and α-synuclein dysfunction potentiate each other, driving chronic progression of neurodegeneration in a mouse model of Parkinson’s disease. *Environ. Health Perspect.* 119 807–814. 10.1289/ehp.1003013 21245015 PMC3114815

[B11] Glass C. K. Saijo K. Winner B. Marchetto M. C. Gage F. H. (2010). Mechanisms underlying inflammation in neurodegeneration. *Cell* 140 918–934. 10.1016/j.cell.2010.02.016 20303880 PMC2873093

[B12] Hickman S. Izzy S. Sen P. Morsett L. El Khoury J. (2018). Microglia in neurodegeneration. *Nat. Neurosci.* 21 1359–1369. 10.1038/s41593-018-0242-x 30258234 PMC6817969

[B13] Hong S. H. Cho J. G. Yoon K. J. Lim D. S. Kim C. H. Lee S. W. (2015). The antibody atliximab attenuates collagen-induced arthritis by neutralizing AIMP1, an inflammatory cytokine that enhances osteoclastogenesis. *Biomaterials* 44 45–54. 10.1016/j.biomaterials.2014.12.017 25617125

[B14] Ismail R. Parbo P. Madsen L. S. Hansen A. K. Hansen K. V. Schaldemose J. L. (2020). The relationships between neuroinflammation, beta-amyloid and tau deposition in Alzheimer’s disease: A longitudinal PET study. *J. Neuroinflammation* 17:151. 10.1186/s12974-020-01820-6 32375809 PMC7203856

[B15] Jung H. J. Park S. H. Cho K. M. Jung K. I. Cho D. Kim T. S. (2020). Threonyl-tRNA synthetase promotes T Helper Type 1 cell responses by inducing dendritic cell maturation and IL-12 production via an NF-κB Pathway. *Front. Immunol.* 11:571959. 10.3389/fimmu.2020.571959 33178197 PMC7592646

[B16] Kim D. G. Lee J. Y. Kwon N. H. Fang P. Zhang Q. Wang J. (2014). Chemical inhibition of prometastatic lysyl-tRNA synthetase-laminin receptor interaction. *Nat. Chem. Biol.* 10 29–34. 10.1038/nchembio.1381 24212136 PMC4021855

[B17] Kim E. Kim S. H. Kim S. Kim T. S. (2006). The novel cytokine p43 induces IL-12 production in macrophages via NF-kappaB activation, leading to enhanced IFN-gamma production in CD4+ T cells. *J. Immunol.* 176 256–264. 10.4049/jimmunol.176.1.256 16365417

[B18] Kim E. Kim S. H. Kim S. Cho D. Kim T. S. (2008). AIMP1/p43 protein induces the maturation of bone marrow-derived dendritic cells with T helper type 1-polarizing ability. *J. Immunol.* 180 2894–2902. 10.4049/jimmunol.180.5.2894 18292511

[B19] Kim M. S. Lee A. Cho D. Kim T. S. (2019). AIMP1 regulates TCR signaling and induces differentiation of regulatory T cells by interfering with lipid raft association. *Biochem. Biophys. Res. Commun.* 514 875–880. 10.1016/j.bbrc.2019.05.040 31084930

[B20] Ko Y. G. Park H. Kim T. Lee J. W. Park S. G. Seol W. (2001). A cofactor of tRNA synthetase, p43, is secreted to up-regulate proinflammatory genes. *J. Biol. Chem.* 276 23028–23033. 10.1074/jbc.M101544200 11292833

[B21] Kumar V. (2019). Toll-like receptors in the pathogenesis of neuroinflammation. *J. Neuroimmunol.* 332 16–30. 10.1016/j.jneuroim.2019.03.012 30928868

[B22] Kwon H. S. Park M. C. Kim D. G. Cho K. Park Y. W. Han J. M. (2012). Identification of CD23 as a functional receptor for the proinflammatory cytokine AIMP1/p43. *J. Cell Sci.* 125 4620–4629. 10.1242/jcs.108209 22767513

[B23] Li J. Shui X. Sun R. Wan L. Zhang B. Xiao B. (2021). Microglial phenotypic transition: Signaling pathways and influencing modulators involved in regulation in central nervous system diseases. *Front. Cell. Neurosci.* 15:736310. 10.3389/fncel.2021.736310 34594188 PMC8476879

[B24] Liang D. Halpert M. M. Konduri V. Decker W. K. (2015). stepping out of the cytosol: AIMp1/p43 potentiates the link between innate and adaptive immunity. *Int. Rev. Immunol.* 34 367–381. 10.3109/08830185.2015.1077829 26325028

[B25] Lim H. S. Kim Y. J. Kim B. Y. Park G. Jeong S. J. (2018). The anti-neuroinflammatory activity of tectorigenin pretreatment via downregulated NF-κB and ERK/JNK Pathways in BV-2 microglial and microglia inactivation in mice with lipopolysaccharide. *Front. Pharmacol.* 9:462. 10.3389/fphar.2018.00462 29867470 PMC5954245

[B26] Liu T. Zhang L. Joo D. Sun S. C. (2017). NF-κB signaling in inflammation. *Signal. Transduct. Target. Ther.* 2:17023. 10.1038/sigtrans.2017.23 29158945 PMC5661633

[B27] Park S. G. Choi E. C. Kim S. (2010). Aminoacyl-tRNA synthetase-interacting multifunctional proteins (AIMPs): A triad for cellular homeostasis. *IUBMB Life* 62 296–302. 10.1002/iub.324 20306515

[B28] Park S. G. Kang Y. S. Ahn Y. H. Lee S. H. Kim K. R. Kim K. W. (2002). Dose-dependent biphasic activity of tRNA synthetase-associating factor, p43, in angiogenesis. *J. Biol. Chem.* 277 45243–45248. 10.1074/jbc.M207934200 12237313

[B29] Qin S. Yang C. Huang W. Du S. Mai H. Xiao J. (2018). Sulforaphane attenuates microglia-mediated neuronal necroptosis through down-regulation of MAPK/NF-κB signaling pathways in LPS-activated BV-2 microglia. *Pharmacol. Res.* 133 218–235. 10.1016/j.phrs.2018.01.014 29391237

[B30] Rao K. M. (2001). MAP kinase activation in macrophages. *J. Leukoc. Biol.* 69 3–10. 11200064

[B31] Saijo K. Glass C. K. (2011). Microglial cell origin and phenotypes in health and disease. *Nat. Rev. Immunol.* 11 775–787. 10.1038/nri3086 22025055

[B32] Spencer J. P. Vafeiadou K. Williams R. J. Vauzour D. (2012). Neuroinflammation: Modulation by flavonoids and mechanisms of action. *Mol. Aspects Med.* 33 83–97. 10.1016/j.mam.2011.10.016 22107709

[B33] Streit W. J. Mrak R. E. Griffin W. S. (2004). Microglia and neuroinflammation: A pathological perspective. *J. Neuroinflammation* 1:14. 10.1186/1742-2094-1-14 15285801 PMC509427

[B34] Tang Y. Le W. (2016). Differential roles of M1 and M2 microglia in neurodegenerative diseases. *Mol. Neurobiol.* 53 1181–1194. 10.1007/s12035-014-9070-5 25598354

[B35] Wang W. Y. Tan M. S. Yu J. T. Tan L. (2015). Role of pro-inflammatory cytokines released from microglia in Alzheimer’s disease. *Ann. Transl. Med.* 3:136. 10.3978/j.issn.2305-5839.2015.03.49 26207229 PMC4486922

[B36] Yang I. Han S. J. Kaur G. Crane C. Parsa A. T. (2010). The role of microglia in central nervous system immunity and glioma immunology. *J. Clin. Neurosci.* 17 6–10. 10.1016/j.jocn.2009.05.006 19926287 PMC3786731

[B37] Zhou Z. Sun B. Huang S. Yu D. Zhang X. (2020). Roles of aminoacyl-tRNA synthetase-interacting multi-functional proteins in physiology and cancer. *Cell Death Dis.* 11:579. 10.1038/s41419-020-02794-2 32709848 PMC7382500

[B38] Zhu X. Liu Y. Yin Y. Shao A. Zhang B. Kim S. (2009). MSC p43 required for axonal development in motor neurons. *Proc. Natl. Acad. Sci. U.S.A.* 106 15944–15949. 10.1073/pnas.0901872106 19717447 PMC2747223

