# Supplementary material for: Aminoacyl transfer ribonucleic acid synthetase complex-interacting multifunctional protein 1 induces microglial activation and M1 polarization via the mitogen-activated protein kinase/nuclear factor-kappa B signaling pathway
Source: Front Cell Neurosci. 2022 Sep 7;16:977205. doi: 10.3389/fncel.2022.977205 (PMC9491728; doi:10.3389/fncel.2022.977205)
Supplement: Supplementary file 1 [file Data_Sheet_1.pdf]

Supplementary Material

Supplementary Figures

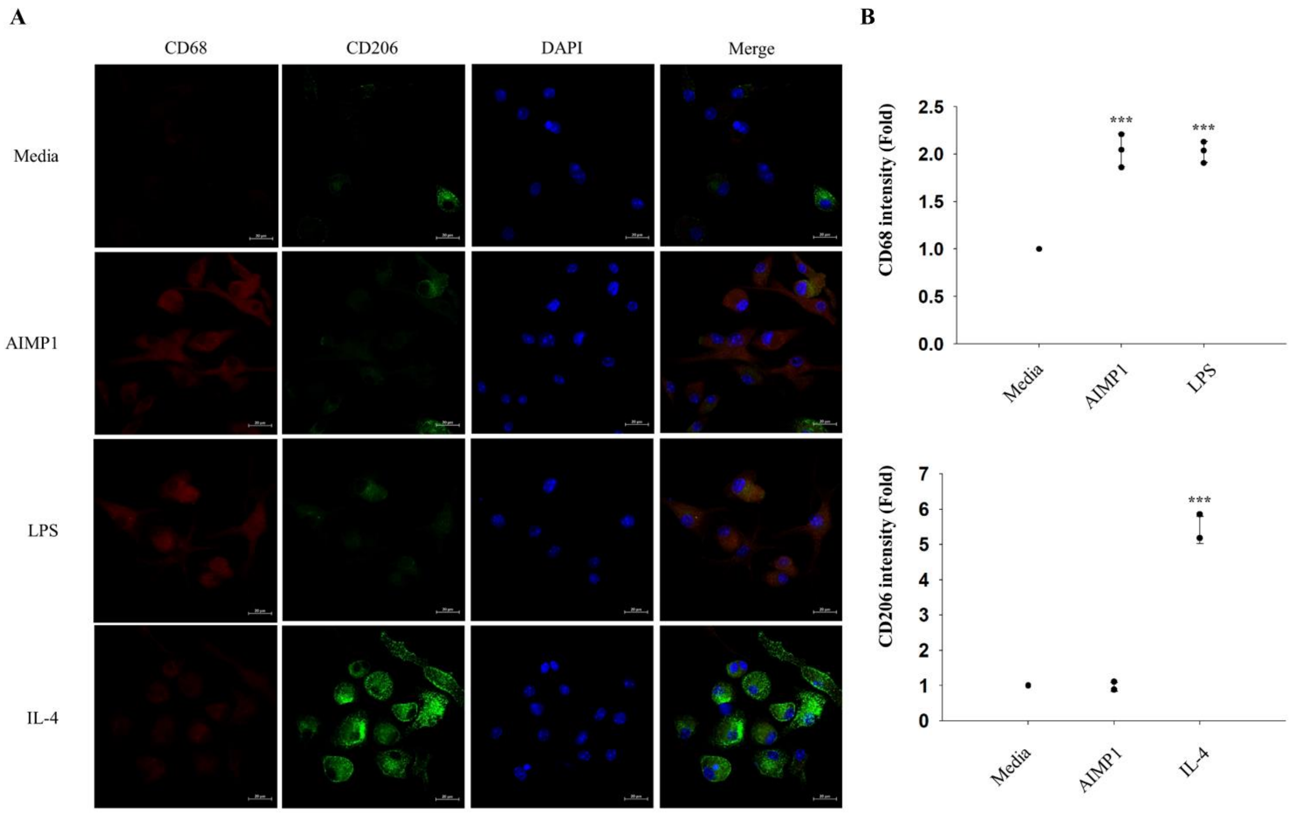

Supplementary Figure 1. AIMP1 induces the polarization of primary microglia into an M1 phenotype but not an M2 phenotype.

(A, B) The primary microglia were treated with AIMP1 (250 nM), LPS (100 ng/mL), or IL-4 (20 ng/mL) for 72 h. **(A)** The expression of CD68 and CD206 was observed using confocal microscopy. All experiments were independently performed thrice, and representative data are shown. **(B)** Scatter plots represent the fold ratio based on the fluorescence intensity of each surface marker in the medium group. The data represent the mean ± SD of three independent experiments. Statistical significance was evaluated using one-way ANOVA with a Bonferroni post-hoc test for multiple comparisons; * $P < 0.05$, ** $P < 0.01$, and *** $P < 0.001$, compared to the medium group.

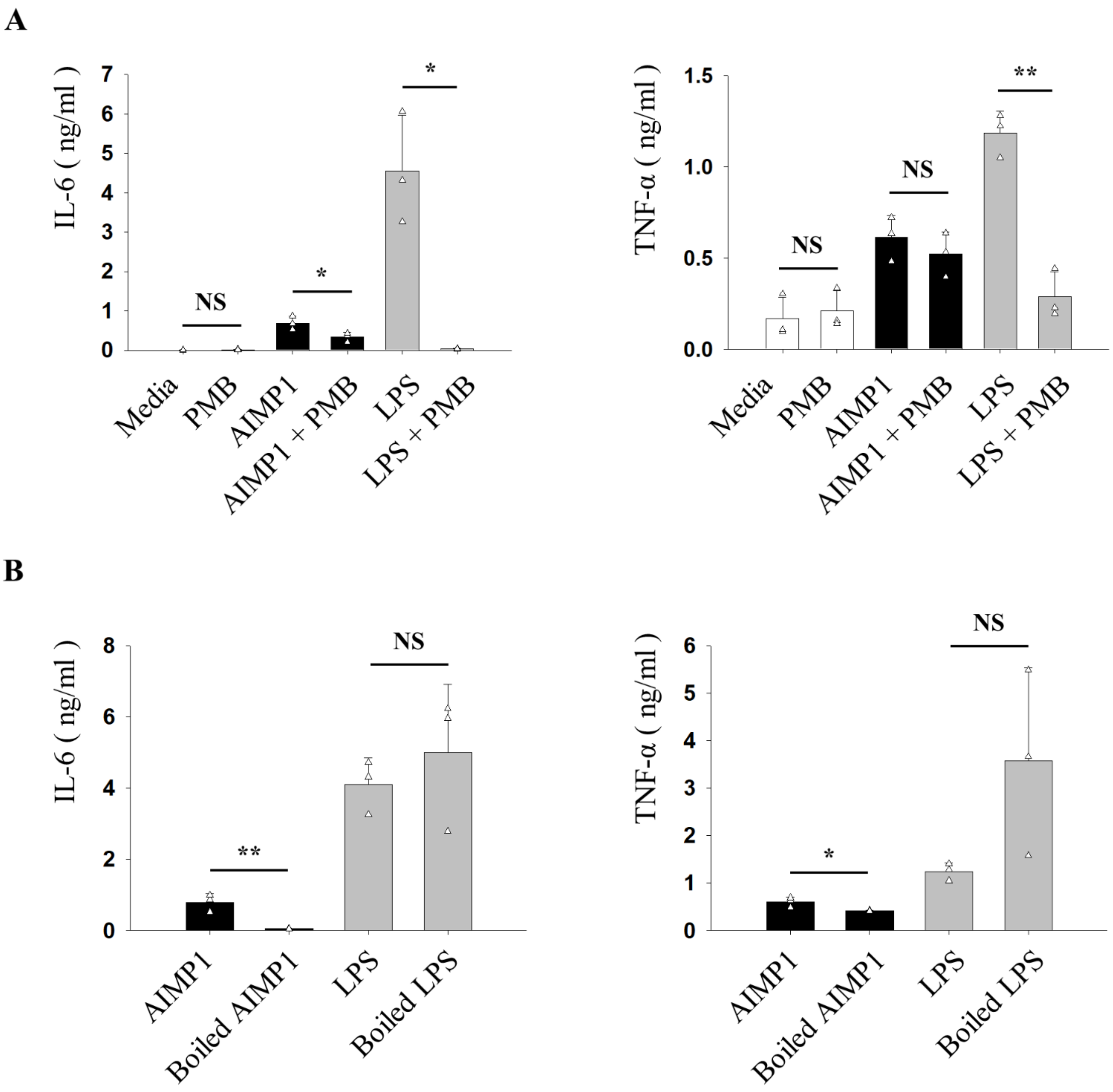

Supplementary Figure 2. AIMP1-induced activation of BV-2 cells is not caused by lipopolysaccharide (LPS) contamination.

(A) BV-2 cells were cultured with AIMP1 (250nM) or LPS (100 ng/mL) in the presence or absence of polymyxin B (PMB) (10 µg/mL) for 24 h. **(B)** BV-2 cells were treated for 24 h with AIMP1 or LPS (100 ng/ml) which was boiled or not. The secretion of IL-6 and TNF-α in the supernatants was determined using ELISA. Bar graphs show the mean ± SD of three independent experiments. Statistical significance was evaluated using an independent Student's *t*-test; ***** P < 0.05, ****** P < 0.01, and ******* P < 0.001.
